# Differences in the Search Behavior of Cancer Detection Dogs Trained to Have Either a Sit or Stand-Stare Final Response

**DOI:** 10.3389/fvets.2020.00118

**Published:** 2020-03-13

**Authors:** Jennifer L. Essler, Clara Wilson, Alexander C. Verta, Rebecca Feuer, Cynthia M. Otto

**Affiliations:** ^1^Penn Vet Working Dog Center, School of Veterinary Medicine, University of Pennsylvania, Philadelphia, PA, United States; ^2^Department of Clinical Sciences and Advanced Medicine, School of Veterinary Medicine, University of Pennsylvania, Philadelphia, PA, United States

**Keywords:** canine, olfaction, biomedical detection, behavior, detection dogs, cancer

## Abstract

Recent literature has demonstrated that dogs have the potential to detect, and communicate the presence of, various human diseases. However, there is a lack of investigation into whether commonplace training differences within the field could influence a dog's behavior during a biomedical detection task. Here we report on the behavior of four dogs trained to alert to blood plasma samples taken from individuals with ovarian cancer. One hundred trials per dog were selected from routine video recordings collected over a period of 13 months. Videos were coded frame by frame to quantify sample checking, alerting behavior, and durations of alert. Dogs had previously been trained to elicit a final response behavior once they had located the target odor. Two dogs had a “sit” response while the other two had a “stand-stare” response. Alert behavior was categorized as true positive (a correct alert to a cancer sample) or false positive (an incorrect alert to biological and non-biological controls and distractors). Hesitations were also recorded, where the dog either checks the sample twice or, spends a longer duration of time sniffing the sample than a true pass without carrying out their final response. Results show individual variation in the total frequency of false alerts elicited. However, the rate of hesitations appears to be influenced by alert style, with stand-stare dogs carrying out 40 and 32, respectively (total = 72) and sit dogs carrying out 7 and 8, respectively (total = 15). The stand-stare dogs had a non-significant difference in the duration of their true and false positive alerts. In contrast, the sit dogs showed a significant difference (*p* < 0.001), maintaining their false alerts for, on average, two times the duration of their true alerts. Stand-stare dogs increased the duration of time spent in contact with the port when plasma samples were present, whereas sit dogs spent on average 0.3 s in contact with the port regardless of what sample type it contained. These findings suggest that the type of operant response a biomedical detection dog has been trained may influence their sample checking and response behavior.

## Introduction

Over the past decade, the use of dogs to detect and alert to human health conditions has expanded. There is growing evidence that dogs can be trained to alert to human disease samples, including, but not limited to: bladder cancer (1), breast cancer (2), cervical cancer (3), colorectal cancer (4), lung cancer (2, 5–7), ovarian cancer (8, 9), prostate cancer (10), melanoma (11), Clostridium difficile (12), and cystic fibrosis bacterial pathogens (13) [see Edwards et al. (14) for the most recent systematic review]. These studies employ a variety of human sample types, including breath, urine, blood plasma, excrement and sebum. Proficient training is fundamental to ensure a dog recognizes their target odor and is motivated to repeat the task over numerous trials. In most cancer detection studies, the dog is further exposed to samples taken from healthy controls, and samples taken from people who have benign tumors. During training, handlers attempt to specify the odor of cancer as the target, as opposed to general human odor or the presence of benign masses, by shaping the dog's response to the cancer positive samples. While individual training methods vary, most dogs are trained using positive reinforcement, with many using the aid of a marker cue (e.g., a clicker) to specify at the precise moment that the dog makes a correct choice (14). If correct, the dog will receive their reward, usually a toy [e.g., (15)] or food [e.g., (16)].

Biomedical detection dogs must be taught two components to be successful. Firstly, dogs must learn their target odor, and be able to discriminate between control and disease positive samples. Secondly, they must be taught a method of communicating that they have located the target odor, known as their “alert.” To communicate with the experimenter, the dogs are conditioned to exhibit a specific behavior, most commonly sitting in front of the target odor. Employment of the sit alert in the biomedical field was likely influenced by passive alerts trained in other working dog fields, such as explosives detection [e.g., (17)]. Of the recent biomedical canine studies published, most reported that dogs had been trained to elicit a sit alert [e.g., (4, 11, 12, 15)]. Jezierski et al. (18) notes that their sampled dogs had a final response dependent on the dog's previous training and the “dog's preference,” however usually consisted of the dog “sitting or lying down in front of the target sample.” While this convention reduces ambiguity for the purposes of the experimenter, it is possible that the arbitrary nature of the behavior may impact their behavior and influence their decisions. It is imperative to minimize factors that may skew a dog's response on such a sensitive odor discrimination task to ensure that response behaviors are driven by the odor source rather than environmental variations. This highlights a potential issue in biomedical detection dog training, where the required alert behavior may actually impact a dog's performance at the task. Mancini et al. (19) highlight this issue, and argue that binary options (e.g., perform the trained alert behavior or do not perform the trained alert behavior) may limit the reliability of a canine's response to a sample. Mancini et al. (19) suggest an “honest signaling” method whereby trained alerts are not implemented, and instead the duration of non-trained behaviors, such as duration of sniffing the port, is used to distinguish between samples. This method, however, relies on the use of technology to accurately track behaviors to the millisecond and would be impossible for a trainer to reliably carry out by eye. Currently, most laboratories still rely on a behavioral cue from the dog to signal detection of the target odor.

The stand-stare alert, whereby the dog remains standing with their nose over the port and freezes, has been less widely used in the current biomedical detection literature. It is possible that dogs who carry out a stand-stare alert may receive more feedback from a sample as they are required to keep their nose on the sample as a function of their alert. Unlike sit alert dogs, to receive their reward, stand-stare dogs must maintain their nose in close proximity to the odor source. Sit alert dogs move back, away from the port, to carry out an alert, which may have an effect on the duration of their false alerts. This study asks whether the type of trained alert impacts a dog's sample checking and alert behaviors while detecting ovarian cancer from human blood plasma samples.

## Materials and Methods

The protocol was approved by the Institutional Animal Care and Use Committee at the University of Pennsylvania for dogs owned by the university (Protocol #804900).

### Videos

Videos were pseudorandomly sampled from Penn Vet Working Dog Center's ovarian cancer detection program archives. Dogs in this program are routinely tested and video recorded in one to four sessions per week using the training protocol described in section Training Protocol. A Canon VICIA HF R700 camera, positioned on a wall mount, recorded all sessions. Videos were included under the restriction that the session had to have taken place once that dog had task acquisition (e.g., not during odor imprinting or alert development stages). Ten recorded sessions were selected per dog, representing 100 trials each. The videos sampled dated from between 08/12/2017 and 11/26/2018.

### Subjects

Dogs included in the study were three females and one male, all neutered or spayed. Breeds were two German Shepherds, one Labrador Retriever and one English Springer Spaniel (min age: 2 years, max age: 7 years, mean age: 4.5 years). Dogs had been taught their alert behavior starting when they arrived at the center, at ~8 weeks of age, and had been imprinted on ovarian cancer blood plasma a minimum of 3 months prior to when the study videos were recorded.

### Training Protocol

**Figure 1:**
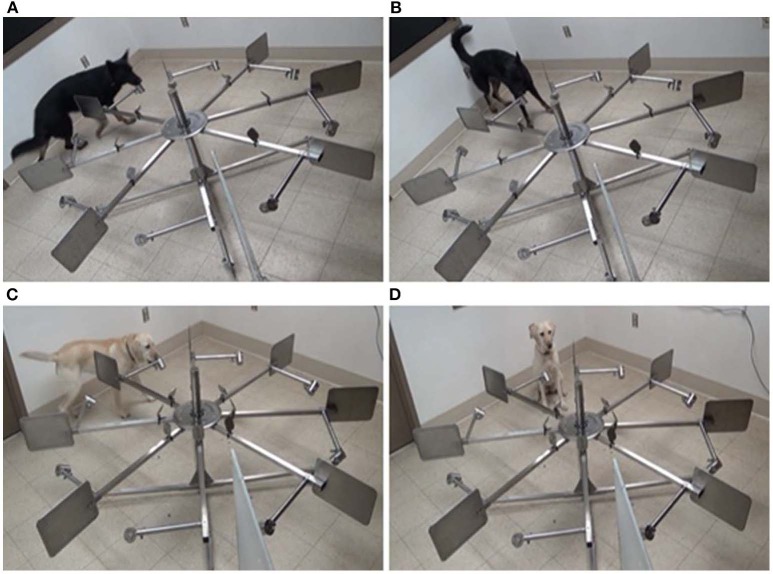
**(A)** Bobbie checking port four (top left). **(B)** Bobbie carrying out a stand-stare alert at port four (top right). **(C)** Ffoster checking port four (bottom left). **(D)** Ffoster carrying out a sit-alert at port four (bottom right).

As part of an ongoing project, dogs are trained one to four times per week to identify human blood plasma samples taken from an individual with ovarian cancer. Each session is video recorded and data is recorded at the time of the session, tracking which sample is in each port and the medical identification of the human biological samples. Trainers and experimenters are out-of-sight behind a wall for all trials, with the dogs observed on a computer monitor screen via video. Dogs are trained on an eight-armed wheel with a “port” on each arm (Medical Detection Dogs, DEMAND—Design and Manufacture for Disability). Each port denotes a receptacle for one sample (see Figure 1). Within each port there is either (1) blood plasma taken from an individual with confirmed ovarian cancer, (2) blood plasma taken from an individual with a benign ovarian tumor (herein denoted as “benign”, (3) blood plasma taken from a healthy individual (herein described as “normal”), (4) a control (a non-biological substance that is involved in the study process and may interfere with the identification of the target odor, e.g., latex gloves, as these are worn when handling samples), or (5) a distractor (a non-biological, unrelated, object e.g., paper clips). Dogs were presented with 75 μl of blood plasma during imprinting, and 50 μl in all subsequent training. For each “hot” trial, there is one cancer sample present (the target odor), and up to two benign or normal samples, the remaining ports contain distractors or controls. For one dog (McBaine, sit alert), an older version of the wheel that has twelve ports was being used at the time of recording (Anne B Kingsley Wheel). The distribution of and quantity of biological samples was identical, with the additional ports being used for additional distractor objects. For all dogs, each session contained ten trials, with 30-50% of these trials being “blanks” (no cancer sample is present). In blank trials, dogs are expected to check all ports of the wheel, and then walk to a raised platform to signal that there is no target odor present. During blank trials, normal, benign, control and distractor samples are present in the wheel.

Every dog was imprinted on the target odor using positive reinforcement and a clicker to mark their correct response. McBaine and Ffoster were shaped to elicit sit response during initial training at the center, and this was taken forward in the rest of their training, including ovarian cancer detection. During imprinting, the cancer odor was presented and the dog would sniff the sample, then told “sit.” This was repeated until the verbal sit command could be phased out and the dog offered it automatically on smelling the cancer sample. Bobbie and Osa were shaped to have a stand-stare alert. This was trained by initially clicking as soon as they sniffed the cancer sample, then building up the duration of the nose-on-port behavior until a full stand-stare was established. During training, dogs were rewarded using either food or a toy, dependent on their preference. Once the target odor could be correctly identified on the wheel among non-biological odors (distractors and controls), other human biological samples were added; first normal samples and then benign samples. Dogs proceeded to each stage of training once they had reached a criterion of 80 percent of trials per session correct over three consecutive days. Videos were only included in the present study once the dogs had reached the final stage. This was carried out to safeguard from potential influences on behavior during the dog acquiring the task.

During all trials utilized for the present study, the dogs were sent to the scent wheel out-of-sight of the trainer and experimenters. The dog searched the wheel while the trainer watched on the computer monitor, and once the dog gave a correct alert on the cancer odor or correctly indicated that the wheel was free of cancer by moving to the raised platform, the trainer marked with a “click” and the dog came out for its reward, either food or a toy. Prior to the investigation into this study, there was no requirement specifically for the length of alert duration required from each dog for stand-stare dogs, and the duration decisions were left to the dog's specific trainer. Similarly, sit dogs were not required to hold a sit beyond it being a clear change of behavior on their target odor.

### Coding

Videos were coded using The Observer XT 14. Behaviors included in the ethogram were based on a dog's response to each port and their alert behaviors (see Table 1). To ensure consistency of coding between alert types, alert behaviors were coded only once the dog had stopped motion. It was important to initiate coding of a sit alert once the dog's haunches touched the ground and the dog became motionless, to exclude the time taken for the dog to go from standing to sitting that would, by default, make the alert time longer. By the same measure, stand-stare alerts were initiated only once the dog had “frozen,” and ended as soon as the dog moved out of their static position (see Figure 1).

**Table 1:** Behavioral ethogram used to code the videos.

**Variable name**	**Description**	**Modifier**	**Measure**
Pass	Dog checks port by making contact with their nose. Dog does not carry out alert behavior or hesitation and instead moves onto the next port or raised platform.	Sample type	Frequency
Hesitate	The dog maintains contact with the port for a greater duration of time than a true pass, but does not carry out their final response. Or, the dog passes the sample then flicks their head back to check the sample a second time.	Sample type	Frequency
Contact with port	Dog puts nose in contact with port.	Sample type	Duration
Stand-stare alert	Dog stands still with nose in contact with, or within one centimeter of, the port. Start behavior when the dog freezes. End behavior when the dog moves their head or body.	Sample type	Duration
Sit alert	Dog checks port and then sits behind port. Start behavior when dog's haunches touch the ground and all movement stops. End behavior when dog moves their head or body.	Sample type	Duration

### Statistical Analysis

Twenty percent of trials were double coded. Inter-rater reliability was assessed using The Observer XT 14 Reliability Analysis function. Data was extracted from The Observer XT to Microsoft Excel version 16.25 for formatting. For each session, the dogs' duration data were averaged such that there was one number accounting for their duration of each behavior (Table 1). Statistical analyses were carried out on R version 3.5.1 (20). Using R package lme4 (21) a linear mixed effect model was run for mean duration of true and false positive alert with alert behavior (sit vs. stand-stare) and alert type (true positive vs. false positive) as fixed effects with an interaction, and dog name as a random effect. MASS package for R (22) was used to carry out generalized linear mixed effects model to compare duration of contact with port. A *p*-value of < 0.05 was considered statistically significant across all tests.

## Results

Inter-rater reliability was above 84% for each session, with an average of 87.73% agreement between observers (Kappa = 0.85, *p* < 0.001). All data can be found in Supplementary Table 1.

### Rates of False Alerts and Hesitations

Over 200 trials (100 trials per dog), the sit dogs elicited a total of 78 false alerts (Ffoster: 41, McBaine: 37) and the stand-stare dogs a total of 48 false alerts (Bobbie: 34, Osa: 14). The sit dogs hesitated on samples only 15 times (Ffoster: 8, McBaine: 7) whereas the stand-stare dogs hesitated a total of 72 times (Bobbie: 42, Osa: 30).

### Duration of True Positive and False Positive Alerts

**Figure 2:**
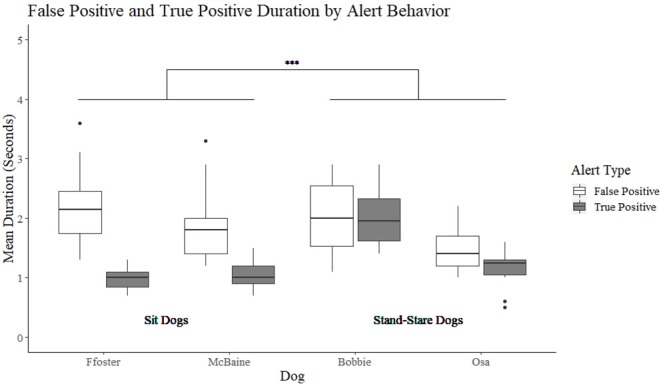
The duration of the sit and stand-stare dogs' true positive and false positive alerts. ***Indicates a significant difference at *p* < 0.001.

A significant interaction was found between alert behavior (sit vs. stand-stare) and alert type (false positive vs. true positive) (*t* = 4.07, *p* < 0.001). The model was further split to compare true and false positive durations between alert behavior group (sit or stand-stare). A non-significant difference was found between the stand-stare dog's mean duration of true and false positive alerts (*t* = −1.24, *p* = 0.223). Bobbie had a mean duration of 2 s for true alerts (min = 0.5, max = 3.7 s), and 2 s for false alerts (min = 0.5, max = 3.3 s). Osa's true positive alerts were on average 1.1 s (min = 0.3, max = 2.1 s) and false positive alerts were 1.4 s (min = 0.8, max = 2.3 s). Conversely, sit dogs showed a significant difference in the duration of their true positive alerts as compared to their false positive alerts (*t* = −7.179, *p* = < 0.001) (Figure 2). Foster had a mean duration of 1 s for true positive alerts (min = 0.4, max = 1.5), and 2.3 s for false positive alerts (min = 1.3 s, max = 6.2 s). McBaine's true positive alerts were on average 1.1 s (min = 0.5, max = 3.1 s) and false alerts on average 2 s (min = 1.4, max = 4.1 s).

### Duration of Contact With Port

**Figure 3:**
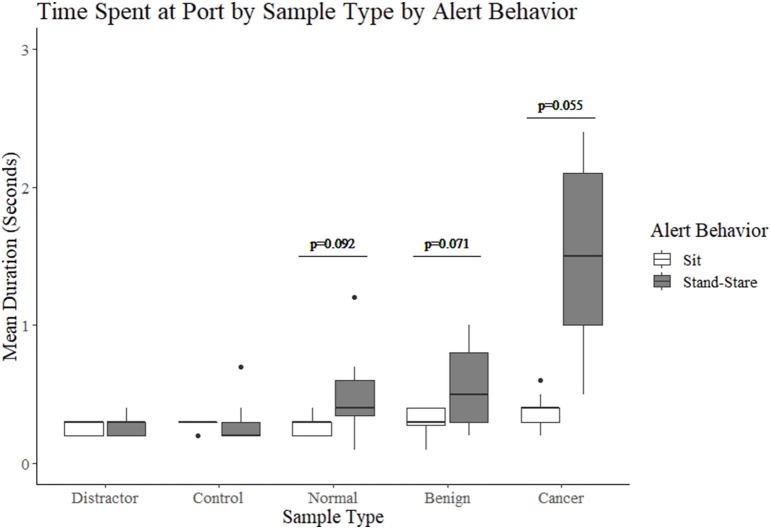
The duration of contact with the port for sit and stand-stare dogs per sample type.

Dogs that show a sit alert spent on average 0.3 s in contact with the port, regardless of whether it contained a distractor, control, normal, benign or cancer sample (Figure 3). In contrast, dogs in the stand-stare group showed an increase in the duration spent in contact with the port, with a mean duration of 0.3 on non-human odor samples (distractors and controls), 0.5 s on normal samples, 0.6 s on benign samples and 1.5 s on cancer samples. Differences in the mean duration of contact with the port between the sit and stand-stare dogs were approaching significance (Figure 3).

## Discussion

Across 400 trials we see individual variation in the number of false alerts each dog performed. Bobbie (stand-stare) showed a similar number of false alerts to McBaine and Ffoster (sit alert). In contrast, Osa (stand-stare) carried out only 14 false alerts over her 100 trials. Given the small number of dogs sampled, a direct association between alert behavior and a dog's overall ability at this task cannot be made. It should also be noted that Osa had a more extensive training history where benign and normal were present but not rewarded [see (9) for details]. This may have contributed to her increased proficiency at the task overall. Of interest is the differences between sit and stand-stare dogs in their number of hesitations on the samples. Sit dogs showed a total of 15 hesitations over 200 trials, whereas the stand-stare dogs hesitated 72 times. These results suggest that the type of operant behavior required to signal an alert may impact on a dog's behavior while checking samples. While Mancini et al. (19) label hesitations as a “breakdown in communication” it could conversely be interpreted as gathering further information on a sample. For example, Mancini et al. (19) highlight the need for dogs to classify samples as “positive, negative or in-between.” As long as strict criterion for reward marking is maintained (e.g., the dog must “freeze” to signal a final response) then it could be argued that a stand-stare alert allows hesitations on a sample to signify this “in-between” response. While hesitations are inherently ambiguous in terms of classifying the sample, it is also important in such a sensitive discrimination task that the dog can check the sample for as long as necessary to make an informed decision. As Mancini et al. (19) highlight, it is possible that dogs become more focused on performing their learnt behavior than on the stimulus coming from the sample. Perhaps within this argument however there are degrees of effect dependent on what the learnt behavior is (e.g., sit or stand-stare).

It could be assumed that a dog's false positive alert would be approximately the same length as their true positive alert. The duration of a true positive alert will be determined by the trainer, as it ends once the marker cue is given. For example, Bobbie showed the longest true positive alert mean duration (2 s) as her trainer used a criterion that Bobbie must be frozen in a true alert for between 1.5 and 3 s before using the clicker. If there was no effect of alert behavior on alert duration, it would be hypothesized that the dog would merely wait for a period of time approximate to when they usually hear their marker cue (the clicker), then move on if they do not hear the cue. By comparing each dog's false positive alert length to their true positive alert length, we were able to assess whether all dogs showed an approximately equal length of true and false positive, or if there was potential impact of alert type on false alert duration. We find that the stand-stare dogs conformed to this hypothesis, with Osa showing a difference of 0.3 s, and Bobbie a difference of 0 s, between their true and false positive alerts. In contrast, the sit dogs carried out false positive alerts for approximately double the duration of their true positive alerts, even though there was never an effort made by their trainers to increase their sit duration for their true positive alert. This may be influenced by the fact that they are no longer getting feedback from the odor source. It is possible that, because a stand-stare behavior requires a dog to keep their nose on the sample, a stand-stare trained dog can continue to receive information from the sample and may move on more quickly from an incorrect response than a sit alert trained dog who has, in carrying out their alert, created more distance from the sample.

For all four dogs, the mean duration of contact with the port was 0.3 s for non-human odor samples (distractors and controls). The sit dogs maintained this mean duration of 0.3 s across all samples, including human odor, whereas the stand-stare dogs elicited a mean duration of 0.5 s for normal samples, 0.6 s for benign samples and 1.5 s for cancer samples. It is not surprising that, for the stand-stare dogs, the longest duration was seen on cancer samples, as their alert behavior includes them making contact with the port. Of particular interest, however, is the increased duration on benign and normal (healthy control) samples. The stand-stare dogs show an increase of duration of contact when a plasma sample of any type is in the port, which may contribute to the increased number of hesitations seen in this group. Though we did not investigate sniffing rates here, Concha et al. (23) found that sniffing behavior in working detection dogs varied between true negatives and other odors. They found that true negatives saw the least number of sniffs by the dogs, compared to true positives, false positives, and false negatives, which elicited twice the number of sniffs. This initially seems to contradict our findings, where the stand-stare dogs spent more time in contact with the port when the odor was a plasma odor regardless of its cancer status (normal, benign, or cancer-positive), even when the dog left the port, marking a true negative. However, the Concha et al. (23) study investigated detection dogs working on the presence or absence of an odor, without controls of similar odor profiles, as seen in cancer detection dogs comparing blood plasmas of different cancer statuses. Nonetheless, future studies should investigate actual sniffing behavior to determine whether time spent with nose on port, prior to and during an alert, are true indicators of more sniffing.

Stand-stare and sit dogs differ in two main ways. Firstly, the sit dogs have the addition of a chained, arbitrary behavior to add on once they have located the target odor (the sit). Secondly, the sit dogs take their nose off the sample to carry out the alert response. Finally, it is important to consider that the stand-stare is similar to a natural “pointing” behavior seen in many dogs and specifically selected for in some breeds (24). Thus, there may in fact be an advantage to using a more naturalistic behavior, that is often seen in response to odor already, rather than adding an arbitrary sit behavior. To disentangle whether one of these aspects may be influencing a dog's behavior more than the other, future studies may wish to include dogs who carry out sit alerts while keeping their nose on the sample. It is possible that training dogs with a sit alert to either keep their nose on the target odor, resulting in more of a “sit-stare” alert, or to engage in more sniffing behavior, may convey to these dogs the same potential advantages seen by the stand-stare dogs in this study.

While this study cannot disentangle whether the results are most influenced by the addition of the unnatural sit behavior or a by-product of their alert including them taking their nose off the sample, the reduced time spent checking each sample regardless of type indicates that perhaps the mere anticipation of carrying out a behavior which involves taking their nose off the port reduces the duration of time spent checking. Given the sensitive nature of the task and the low odor thresholds involved (up to parts per trillion), it may be most prudent to employ a system which does not limit a dog's interaction with the sample, such as training an alert which involves them moving away from the sample itself. While arguably ambiguous, hesitations may, in fact, further provide more information on a sample that a binary pass/alert response would fail to communicate. In training a stand-stare alert, it is important to establish a “freeze” to mark out the final response behavior. In doing so, the dog is able to check the sample for a greater amount of time prior to making their final response. The results of this study indicate that a stand-stare alert may facilitate this process to a greater extent than an operant response that involves the dogs moving off the sample.

It must be considered that these results were carried out on a limited sample of dogs. This is unfortunately a field-wide issue, as multiple laboratories test different human diseases, often with limited access or resources to train a sizeable sample of dogs. For example, several articles in this field offer important proof-of-principle data, but involve only a single canine [e.g., (12, 15)]. A lack of access to a large sample of trained dogs limits the scope to assess aspects such as alert behavior on task performance. Previous research has shown that individual characteristics of dogs' impact on their accuracy on human disease detection tasks [e.g., (25)], therefore a larger sample size would be needed to corroborate that these findings are related to the alert type rather than individual differences. However, results within the two “sit-stare” dogs were consistent to each other, and similarly results within the two “stand-stare” dogs, suggesting that there were effects of alert style as opposed to random variation between individual dogs. To compensate for the limited access to a wider pool of trained dogs, a larger number of trials per dog was chosen to establish robust findings within-dog. If these preliminary results can be established on a larger sample of trained dogs, there could be important applications to the field.

It is currently commonplace to allow the dog's preference to guide their final alert behavior, as it was previously thought that, within operant trained responses, alert type does not impact task behaviors. The results of this study indicate otherwise. While passive alerts may be ideal in other detection dog roles, for example in providing a non-ambiguous response at great distances, in a laboratory setting a sit response may be sub-optimal. Biomedical detection dogs are tasked with comparing multiple odor sources, many with a similar odor profile, in close proximity to one another. In a line-up of eight human samples, where, for example, four are different healthy controls, three are from people with benign tumors and one is a cancer positive sample, the level of specificity needs to be extremely high. When considering further that the dog may be given as little as 50 μl of sample, it may be beneficial to intentionally train an operant response that, by definition, includes the dog keeping their nose on the sample longer. This may allow dogs to make more informed decisions as a product of them having an additional motivation to keep their nose on the sample. It may also reduce the likelihood of the dog making incorrect decisions without the ability to change response because they have moved away from the sample and are no longer able to get feedback from it. It should be considered that both sit and stand-stare alerts are still operant behaviors that need to be shaped and trained in a similar way. However, without the means to use “honest signaling” (e.g., using technology to measure non-trained responses to a sample), a stand-stare alert may offer trainers a more truthful method of communication than a sit response.

## Conclusion

Currently in biomedical detection research a sit alert final response is most commonly used. Until now, it was widely considered that operant alert type would not impact on task-related behaviors. This study suggests that alert type may influence the duration of a dog's false positive alert, and the amount of time spent checking a sample. Individual differences in the total number of false alerts recorded prohibits judgment on whether alert type directly affects task accuracy. Given the potential lack of feedback available once a dog has sat back away from the sample, it is possible that training a stand-stare alert instead may provide more information to the canine and assist in their categorization of the sample.

## Data Availability Statement

All datasets generated for this study are included in the article/Supplementary Material.

## Ethics Statement

The studies involving human participants were reviewed and approved by University of Pennsylvania Institutional Review Board, Approval # 702679. The patients/participants provided their written informed consent to participate in this study. The animal study was reviewed and approved by University of Pennsylvania Institutional Animal Care and Use Committee.

## Author Contributions

JE, CW, AV, RF, and CO planned the study, edited and finalized the manuscript. JE, CW, AV, and RF collected the data. CW and AV analyzed the videos. JE analyzed the data. JE and CW drafted the original manuscript. CO acquired funding for the project.

### Conflict of Interest

The authors declare that the research was conducted in the absence of any commercial or financial relationships that could be construed as a potential conflict of interest.

## Supplementary Material

Supplementary Table 1 Data used for this study.
